# Evaluation and Outcomes of Hearing Loss in Temporal Bone Fractures: A Prospective Study

**DOI:** 10.7759/cureus.46331

**Published:** 2023-10-01

**Authors:** Kiran A Deshmukh, Uzra Fatima, Ayesha Siddiqui, Mallikarjun S Tegnoor

**Affiliations:** 1 Otolaryngology - Head and Neck Surgery, Mahadevappa Rampure Medical College, Kalaburgi, IND

**Keywords:** sensorineural hearing loss, hemotympanum, conductive hearing loss, head trauma, temporal bone fracture

## Abstract

Background

Fractures of the skull base occur in 3-30% of head injury presentations to the emergency department. Overall, 9-40% of the cases have temporal bone fractures (TBFs). This fracture may disrupt the intervening structures causing edema, hematoma, bleeding, hearing loss, dizziness, cerebrospinal fluid otorrhea, and facial nerve paralysis. This study aims to evaluate the type of TBF, its correlation with hearing loss, and the outcomes of hearing loss.

Methodology

A prospective observational study was done among 50 patients who presented to the emergency department following trauma with clinical features and CT of the temporal bone suggestive of TBF. A complete evaluation of the patients was done, and patients were managed as per the departmental protocol. The patients were followed up for six months and monitored for otological symptoms. Periodic assessment of hearing loss by pure tone audiometry (PTA) was performed at the end of one week, one month, and six months.

Results

The most common type of fracture in our study was longitudinal TBF (72%), followed by transverse TBF (20%) and mixed TBF (8%). According to the newer classification, otic capsule-sparing fracture was more common than otic capsule-violating fracture. Most patients presented with conductive hearing loss (60%) following the TBF. On follow-up, there was a statistically significant improvement in hearing loss at the end of six months.

Conclusions

Our study found that in most cases hearing loss improved over time. Patients with conductive hearing loss showed maximum improvement in comparison to patients with sensorineural and mixed hearing loss.

## Introduction

A significant lateral force to the cranium can cause complex injuries of the petrous region resulting in temporal bone fractures (TBFs). These injuries can result from a motor vehicle accident (MVA), high-impact sports, or, less frequently, from trivial falls. The incidence of TBF varies greatly. In polytrauma cases, the incidence has been reported to be 32 per 1,000 cases [1]. TBF has been reported in up to 40% of cases with head injuries [2].

As the temporal bone houses several important structures, TBFs can be complicated by facial nerve palsy, cochlear injury, vestibular organ injury, and injury to the carotid artery/jugular vein. Thus, each case of head injury/polytrauma needs to be evaluated for trauma to the temporal bone as this may be frequently overlooked at the initial point of contact in the emergency department (ED). This may be critical in determining the acute as well as long-term management plan.

This study aimed to determine the incidence, clinical presentations, types, and outcomes of hearing loss in different types of TBFs.

## Materials and methods

This prospective observational study was conducted in a tertiary care hospital of Mahadevappa Rampure Medical College (MRMC) in the Department of Otorhinolaryngology. This study was conducted over 18 months from March 2021 to August 2022. Ethics approval (approval number: HKES/MRMCK/IEC/210248) was granted by the MRMC Institutional Ethics Committee before recruitment. All participants provided written informed consent.

Patients and data collection

Patients irrespective of their age group with a high-resolution computed tomography (HRCT)-diagnosed/confirmed TBF were included in this study. All included patients were either directly referred to our department from the ED or were subsequently referred from the Department of Neurosurgery or Oral and Maxillofacial Surgery. Patients with a history of any hearing loss and neurological insults (e.g., stroke, tumors, facial nerve palsy, head trauma) were excluded from the study.

Case history was noted detailing the specifics of the trauma (mode, cause, and mechanism) and clinical symptomatology. A detailed clinical evaluation was performed including a general physical, otological, and neurological examination including House Brackman’s staging for facial nerve palsy if relevant. The demographic details were also collected.

In HRCT, serial 1 mm thick sections, 120 kV, 140 mA, 1 mm interval, 0.8 mm nominal single collimation width and 1.6 mm total beam collimation width, and 0.562:1 pitch were obtained in both axial and coronal planes. Axial images were obtained parallel to the orbitomeatal plane. Coronal sections were obtained at a scanning angle parallel to the vertical ramus of the mandible.

Traditional TBF classification system was followed in this study, namely, longitudinal, transverse, and mixed fractures, based on the orientation of the fracture line relative to the long axis of the petrous pyramid [3,4]. An alternative system considering fractures as the otic capsule-sparing (OCS) or otic capsule-violating (OCV) is becoming more widely adopted. Rather than just describing the fracture orientation this system emphasizes the structures involved in the fracture. In accordance with the new system, TBFs were also classified as OCV fractures involving the petrous bone, i.e., the otic capsule and the petrous apex, and as OCS fractures sparing the petrous bone but involving the external auditory canal (EAC), the middle ear (ME), and/or the mastoid [2,5,6].

**Figure 1:**
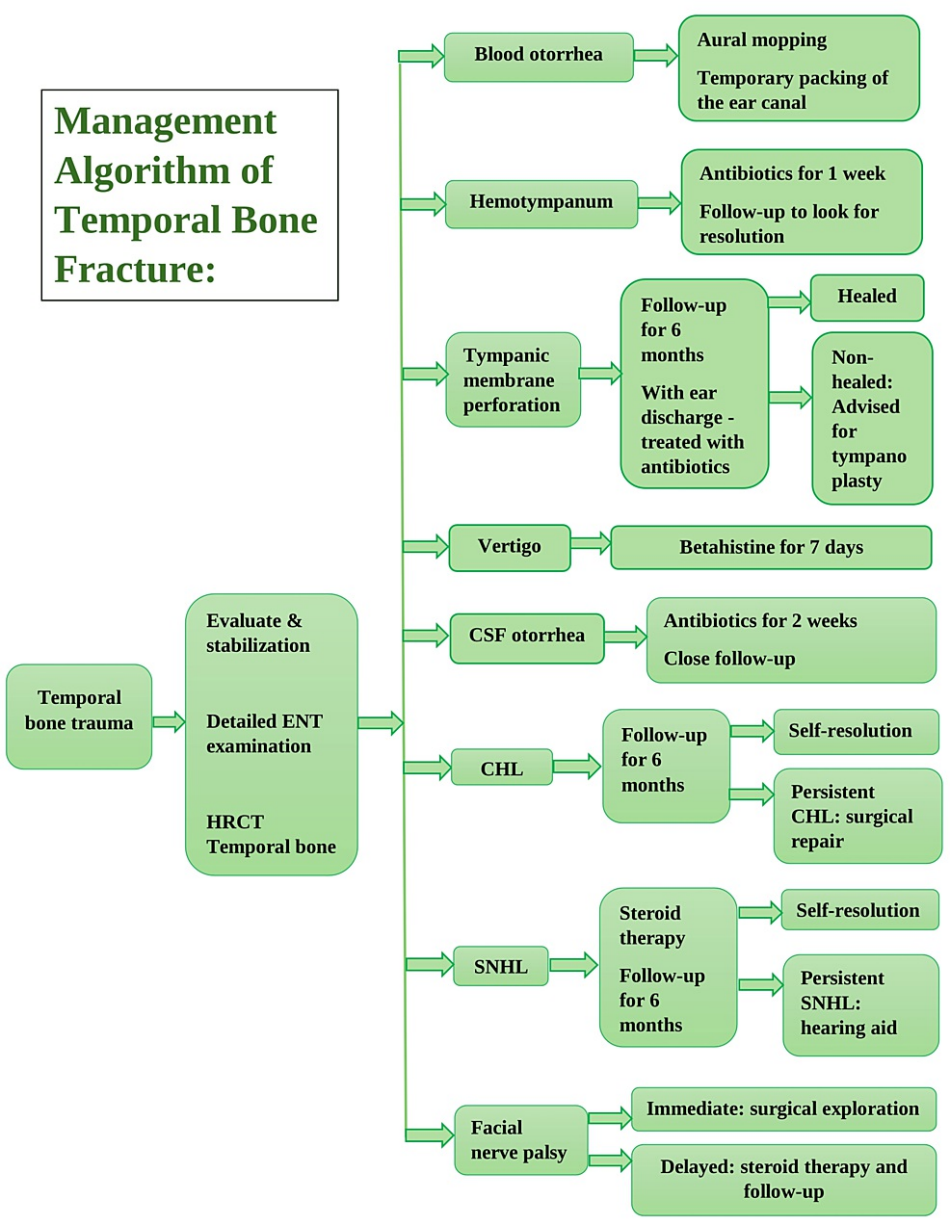
Algorithm for the management of all patients with temporal bone fractures. CHL: conductive hearing loss; CSF: cerebrospinal fluid; ENT: ear, nose, and throat; HRCT: high-resolution computed tomography; SNHL: sensorineural hearing loss

We developed an algorithm for the management of all patients with TBFs to standardize and deliver a consistent line of care (Figure 1).

Outcomes

The primary outcome assessed was hearing loss. For up to the initial six months, patients were followed up and monitored for otological symptoms. Hearing loss was assessed by performing a pure tone audiogram (PTA) at the end of the first week, first month, and sixth month. Secondary outcomes included the clinical presentation and any complications such as tympanic membrane perforation, hemotympanum, facial nerve palsy, or cerebrospinal fluid (CSF) otorrhea.

Statistical analysis

Data was analyzed using SPSS version 25.0 (IBM Corp., Armonk, NY, USA) software. For quantitative data analysis of descriptive statistics, mean and standard deviation were calculated initially. Independent-sample t-test was used to compare the mean values between two variables for statistical significance. For qualitative data analysis, the chi-square test and Fisher exact probability tests were applied for statistical significance. P-values ≤0.05 were considered statistically significant for all comparisons.

## Results

**Figure 2:**
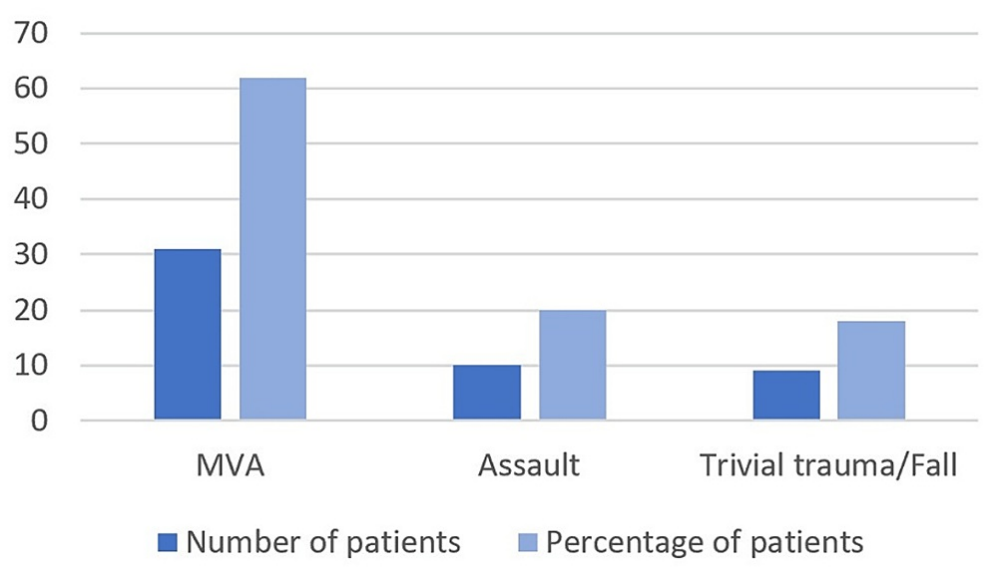
Mode of injury causing temporal bone fractures. MVA: Motor vehicle accident

A total of 50 patients met the inclusion criteria of HRCT-diagnosed TBF. The age range of the included patients was 12-65 years, and the mean age was 32 (±15) years. There was a male preponderance (80%) in our cohort with a male-to-female ratio of 4:1. MVAs were the most common mode of injury seen in 31 patients (62%) (Figure 2). Among the 50 patients with TBFs, the incidence rate of hearing loss was 80%, i.e., 10 patients had normal hearing while 40 patients had some degree of hearing loss. The baseline characteristics of the study participants are summarized in Table 1.

**Table 1:** Baseline demographic and mode of injury data.

Age	Number (%)
<18 years	6 (12)
18–60 years	38 (76)
>60 years	6 (12)
Sex distribution	
Male	40 (80)
Female	10 (20)
Mode of injury	
Motor vehicle accident	31 (62)
Assault	10 (20)
Self- fall	9 (18)

According to the traditional classification, 36 were classified as longitudinal, 10 as transverse, and four as mixed TBFs. Of the 36 longitudinal TBFs, four were bilateral while the remaining 32 were unilateral TBFs. According to the newer classification system of TBFs, 42 (84%) were OCS fractures while eight (16%) were OCV fractures (Table 2).

**Table 2:** Distribution of temporal bone fractures as per the traditional and new system of classification.

	Number (%)
Traditional classification system	
Longitudinal	36 (72%)
Transverse	10 (20%)
Mixed	4 (8%)
Newer system of classification	
Otic capsule-sparing (OCS)	42 (84%)
Otic capsule-violating (OCV)	8 (16%)

In our cohort of TBFs, ear bleeding was found to be the most common symptom at presentation to the ED seen in 41 (82%) patients. Reduced hearing was the second most common symptom at presentation seen in 24 (48%) patients, followed by otalgia in 18 (36%) patients, and watery ear discharge in 4 (8%) patients. Other rarer symptoms included giddiness in 8 (16%) and tinnitus in three (6%) patients. Facial palsy was present in two patients at presentation (Table 3). On a detailed otological examination, active bleeding was seen in the EAC of 41 (82%) patients. Tympanic membrane perforation was seen in 11 (22%) patients. Hemotympanum was found in 20 (40%) patients (Table 3).

**Table 3:** Otological clinical features at presentation to the emergency department. EAC: external auditory canal; TM: tympanic membrane

Clinical symptoms	Number (%)	Clinical signs	Number (%)
Ear bleed	41 (82)	Active ear bleed	41 (82)
Reduced hearing	24 (48)	TM Perforation	11(22%)
Earache	18 (36)	Hemotympanum	20 (40)
Giddiness	8 (16)	Pinna injury	11 (22)
Watery ear discharge	4 (8)	Blood clots in the EAC	8 (16)
Tinnitus	3 (6)	Watery otorrhea	4 (8)
Facial palsy	2 (4)		

On follow-up, at the end of the first week, the most common otological finding was hemotympanum seen in 20 cases, which is one of the causes of transient conductive hearing loss. At the end of the first month, hemotympanum was seen in 13 cases, and by the end of six months, hemotympanum had resolved in all cases. Traumatic tympanic membrane perforation was seen in 11 cases at the end of the first week. On follow-up at the end of the first month, two patients had a healed tympanic membrane, and perforation was seen in nine cases. At the end of six months, eight patients had tympanic membrane perforation and three had healed tympanic membrane. At the end of six months, eight cases had persistent tympanic membrane perforation.

Among the 50 patients with TBFs, 10 patients had normal hearing and the number of patients with hearing loss was 40.

At the end of the first week, the distribution of the different types of hearing in different types of TBFs as per the two classification systems is detailed in Table 4. There was a statistically significant correlation between types of TBF with the type of hearing loss at the end of one week (p < 0.001) as per the traditional (p = 0.000) and OCS/OCV (p = 0.007) classification system. On follow-up at the end of the first month, there was a statistically significant correlation between types of TBF with the type of hearing loss (p < 0.001) as per the traditional (p = 0.001) and OCS/OCV (p = 0.028) classification system. On follow-up at the end of six months, there was a statistically significant correlation between types of TBF with the type of hearing loss (p < 0.001) as per the traditional (p = 0.003) and OCS/OCV (p = 0.004) classification system.

**Table 4:** Correlation of the classification of temporal bone fractures with hearing loss on follow-up at the end of one week, one month, and six months. CHL: conductive hearing loss; MHL: mixed hearing loss; OCS: otic capsule-sparing; OCV: otic capsule-violating; SNHL: sensorineural hearing loss; S: significant; HS: highly significant; VHS: very highly significant

At the end of the first week							
Type of hearing loss	Traditional classification			P-value	OCS/OCV classification		P-value
	Longitudinal (n = 27)	Transverse (n = 9)	Mixed (n = 4)		OCS (n = 32)	OCV (n = 8)	
CHL (24)	23 (85.2%)	1 (11.1%)	0 (0.0%)	P = 0.000, VHS	24 (75.0%)	0 (0.0%)	P = 0.007, HS
SNHL (8)	2 (7.4%)	5 (55.5%)	1 (25.0%)		3 (9.4%)	5 (62.5%)	
MHL (8)	2 (7.4%)	3 (33.3%)	3 (75.0%)		5 (15.6%)	3 (37.5%)	
At the end of the first month							
	Longitudinal (n = 15)	Transverse (n = 8)	Mixed (n = 4)	P-value	OCS (n = 19)	OCV (n = 8)	P-value
CHL (15)	13 (86.6%)	1 (12.5%)	1 (25.0%)	P = 0.001, VHS	13 (68.4%)	2 (25.0%)	P = 0.028, S
SNHL (6)	1 (6.7%)	4 (50.0%)	1 (25.0%)		3 (15.8%)	3 (37.5%)	
MHL (6)	1 (6.7%)	3 (37.5%)	2 (50.0%)		3 (15.8%)	3 (37.5%)	
At the end of the sixth months							
	Longitudinal (n = 8)	Transverse (n = 6)	Mixed (n = 3)	P-value	OCS (n = 11)	OCV (n = 6)	P-value
CHL (8)	7 (87.5%)	0 (0.0%)	1 (33.3%)	P = 0.003, HS	7 (68.4%)	1 (25.0%)	P = 0.0047, S
SNHL (4)	0 (0%)	3 (50.0%)	1 (33.3%)		2 (15.8%)	2 (37.5%)	
MHL (5)	1 (12.5%)	3 (50.0%)	1 (33.4%)		2 (15.8%)	3 (37.5%)	

**Figure 3:**
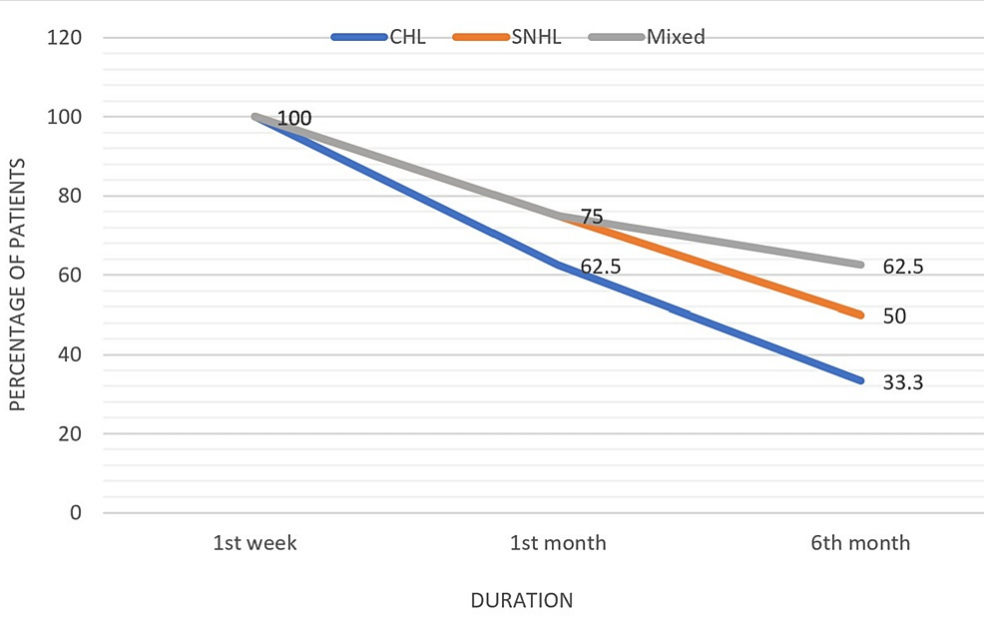
Graph showing the decrease in the number of cases of all types of hearing loss. CHL: conductive hearing loss; SNHL: sensorineural hearing loss

On follow-up, we observed that all the types of hearing loss improved with time. Our study showed a statistically significant reduction of conductive hearing loss (p = 0.008), sensorineural hearing loss (p = 0.017), and mixed hearing loss (p = 0.025) with time (Table 5, Figure 3).

**Table 5:** Improvement of hearing loss in temporal bone fractures. CHL: conductive hearing loss; MHL: mixed hearing loss; SNHL: sensorineural hearing loss; HS: highly significant; S: significant

Type of hearing loss	Duration			P-value
	First week	First month	Sixth month	
	N (%)	N (%)	N (%)	
CHL	24 (100.0%)	15 (62.5%)	8 (33.3%)	0.008, HS
SNHL	8 (100.0%)	6 (75.0%)	4 (50.0)	0.017, S
MHL	8 (100.0%)	6 (75.0%)	5 (62.5%)	0.025, S

## Discussion

TBFs are caused by a significant lateral force to the cranium. A tremendous force is needed to cause TBF; hence, the higher incidence of TBF is directly proportional to the severity of the force of collision in MVAs.

According to the literature, TBFs are commonly seen in male gender, younger age groups, and in MVAs. In our study, MVA was the most frequent cause, followed by other causes such as assault and a fall from a height. There was a male preponderance in our study, with a male-to-female ratio of 4:1. Mechanism of injury and sex distribution in our study were comparable to studies by Bhindi et al. [7] and Montava et al. [8]. On presentation to the ED, the most common presenting symptom was ear bleed seen in 41 (82%) patients while 24 (48%) patients complained of reduced hearing. Active bleeding in the EAC was seen in 41 (82%) cases, hemotympanum in 20 (40%) cases, blood clots in the EAC in nine (18%) cases, and watery otorrhea was seen in four (8%) cases. Similar findings were reported in the study by Montava et al. [8] where 34 (79.1%) patients had otorrhagia, five (11.6%) had a CSF leak, 18 (41.9%) had an external acoustic meatus wound, 26 (60.5%) had a tympanic membrane perforation, and 37 (86%) had hemotympanum.

Among 50 patients included in our study, the most common fracture was longitudinal TBF seen in 36 (72%) patients, 10 (20%) had transverse TBF, and four (8%) had mixed TBF. As per otic capsule involvement, 42 (84%) had OCS fractures while eight (16%) had OCV fractures. Studies by Prasad et al. [9] and Padmakumar et al. [1] also found OCS to be much more common than OCV fractures.

All our patients were followed up for six months and were evaluated clinically. In the study conducted by Maradi et al. [10], tympanic membrane perforation was present in only 18 (40%) cases. Among these, 13 (72.3%) had small-sized perforations while five (27.3%) had medium-sized perforations in the pars tensa. Only 10 cases had persistent perforation by the end of the fourth week. By the 12th week, this had reduced by 66.7%, with only three cases having a persistent, small-sized traumatic perforation. In our study hemotympanum had resolved in all 20 of our cases. Tympanic membrane perforation had healed in only three cases while the remaining eight had a perforated tympanic membrane. Endoscopic repair of the tympanic membrane has advantages over the microscopic tympanoplasty [11]. Resolution of hemotympanum and spontaneous healing of the tympanic membrane was probably the reason for such significant improvement in conductive hearing loss on follow-up at six months.

In our study, all four (8%) cases of CSF leaks had resolved with conservative management in about a week. CSF leaks generally heal in two weeks. CSF leaks that persist longer than 10-14 days most likely require surgical repair [1]. The incidence of CSF leaks in our study was similar to the study by Ljilijana et al. [12]. In our study, two patients presented with House-Brackmann grade 3 facial palsy at the time of presentation to the ED. There was no evidence of any bone impinging on the facial nerve on HRCT of the temporal bone. These patients were advised to take oral steroids for two weeks along with physiotherapy. On the first month of follow-up, one patient presented with grade 2 facial palsy, which was completely resolved by the sixth month. One patient had no improvement in facial nerve functions, and he was advised to undergo facial nerve decompression which the patient did not consent to. The available data indicate that conservative, non-operative management is associated with facial nerve recovery in a high percentage of cases of immediate-onset complete facial nerve paralysis. This suggests that even in cases of immediate-onset complete facial paralysis, many patients will have mechanically intact facial nerves capable of recovery from the injury. It is recommended that patients who have acute-onset, complete facial nerve paralysis be reassessed between three and seven days after the injury to allow for Wallerian degeneration to occur.

Conductive hearing loss is the most common type of hearing loss in TBF. This could possibly be due to the presence of blood in the EAC, ossicular disruption, or tympanic membrane perforation. The high rate of hemotympanum and tympanic membrane perforations may be the cause of this high ratio of hearing loss. Among the 17 patients with hearing loss, eight had conductive hearing loss, five had sensorineural hearing loss, and five had mixed hearing. A similar distribution of the type of hearing loss was seen in a study by Abhishek et al. [13]. In another study, the prevalence of conductive hearing loss was 12.3% in patients with hearing loss after an average of four to six weeks of follow-up. After an average of four to six weeks of follow-up, conductive hearing loss often improves with time (usually within three to four weeks) [14]. Pure hemotympanum generally resolves without sequelae within this time period as well. Small tympanic membrane perforation also heals within four to six weeks. Transient conductive hearing loss may be observed up to the resolution of the hemotympanum [15].

The longitudinal fracture traverses the EAC and produces hemotympanum, typically with conductive hearing loss. conductive hearing loss can be due to the hemotympanum itself behind the tympanic membrane, or from tympanic membrane perforation or ossicular chain disruption. Transverse fractures, on the other hand, can compromise the otic capsule and cause profound sensorineural hearing loss. It is possible to sustain direct injuries that disturb the membranous labyrinth or concussions [16]. Sensorineural hearing loss or vestibular dysfunction may occur as a result of labyrinthine concussions, either temporarily or permanently. The symptoms of sudden hearing loss, vertigo, and possible horizontal nystagmus are frequently reported by patients with OCV fractures [17]. Over time, conductive hearing loss typically improves (usually within three to four weeks). A study of hearing loss from TBF found that 80% of cases of conductive hearing loss from a longitudinal fracture resolved spontaneously [16]. Damage to the cochlea, interruption of blood supply to the cochlea, transection of the cochlear labyrinth or the eighth cranial nerve, and concussive events within the scala media have been reported to result in sensorineural hearing loss after a head injury [18]. In our study, at the end of six months, patients with conductive hearing loss showed the maximum improvement in hearing. There was a statistically significant reduction of conductive hearing loss with duration, signifying that there was transient hearing loss, which resolved with time. Thus, early identification of TBF and their detailed evaluation avoids some of the delayed complications of these fractures which may have a strong impact on the quality of life.

Limitations of the study include a small sample size. In the future, a multi-institutional prospective study would yield better quality results. The data derived from such studies may be applicable at the national level to develop a standardized operating protocol for all patients with TBF. A longer follow-up period and more frequent assessment of hearing would have provided a much better picture of the increase/decrease of hearing. The absence of evidence of pre-trauma hearing loss also added to the limitations as our study population included patients more than 60 years of age and may have had some age-related sensorineural hearing loss.

## Conclusions

TBFs occur due to high-intensity trauma resulting in a wide range of otological manifestations. Our study found that among patients with TBFs, conductive hearing loss was more common than sensorineural hearing loss and mixed hearing loss. Our follow-up showed that in most cases hearing loss improved over time. Patients with conductive hearing loss showed significantly better improvement in comparison to patients with sensorineural and mixed hearing loss. Early intervention in the form of surgery is required in a few cases to avoid worsening of hearing loss and poor quality of life.

This study showed that a majority of TBF-associated hearing loss improves over time with conservative management. However, in all cases, neuroimaging plays a crucial role in determining whether active surgical management may be required in some cases for better outcomes.

Further studies are required with a bigger sample size and longer follow-up periods. Studies comparing the two classification systems in terms of the degree, severity, and long-term prognosis of each type of hearing loss are also required.

## References

[1] Padmakumar V, Ramesh Kumar E, Ramakrishnan VR (2020). A prospective study on temporal bone involvement in polytrauma patients and the effect of early diagnosis on hearing loss. Indian J Otolaryngol Head Neck Surg.

[2] Toynton S (2018). Ear trauma. Scott-Brown’s Otorhinolaryngology Head and Neck Surgery.

[3] Johnson F, Semaan MT, Megerian CA (2008). Temporal bone fracture: evaluation and management in the modern era. Otolaryngol Clin North Am.

[4] Rafferty MA, Mc Conn Walsh R, Walsh MA (2006). A comparison of temporal bone fracture classification systems. Clin Otolaryngol.

[5] Dahiya R, Keller JD, Litofsky NS, Bankey PE, Bonassar LJ, Megerian CA (1999). Temporal bone fractures: otic capsule sparing versus otic capsule violating clinical and radiographic considerations. J Trauma.

[6] Varo Alonso M, Utrilla Contreras C, Díez Tascón Á, García Raya PS, Martí de Gracia M (2019). Traumatic injury of the petrous part of the temporal bone: keys for reporting a complex diagnosis. Radiologia (Engl Ed).

[7] Bhindi A, Carpineta L, Al Qassabi B, Waissbluth S, Ywakim R, Manoukian JJ, Nguyen LH (2018). Hearing loss in pediatric temporal bone fractures: evaluating two radiographic classification systems as prognosticators. Int J Pediatr Otorhinolaryngol.

[8] Montava M, Masson C, Lavieille JP, Mancini J, Soussan J, Chaumoitre K, Arnoux PJ (2016). Temporal bone fracture under lateral impact: biomechanical and macroscopic evaluation. Med Biol Eng Comput.

[9] Prasad BK, Basu A, Sahu PK, Rai AK (2022). A study of otological manifestations of temporal bone fractures. Indian J Otolaryngol Head Neck Surg.

[10] Maradi N, Somanath BM (2017). Hearing loss following temporal bone fractures- a study on classification of fractures and the prognosis. Int J Otorhinolaryngol Head Neck Surg.

[11] Deshmukh KA, Kurle V (2020). Endoscopic versus microscopic type 1 tympanoplasty in chronic suppurative otitis media- tubotympanic type. Int J Otorhinolaryngol Head Neck Surg.

[12] Cvorovic L, Jovanovic MB, Markovic M, Milutinovic Z, Strbac M (2012). Management of complication from temporal bone fractures. Eur Arch Otorhinolaryngol.

[13] Abhishek M, Kaleeswaran R, Srinivasan K (2021). Assessment of hearing loss in temporal bone fractures. Indian J Otol.

[14] Yalçıner G, Kutluhan A, Bozdemir K, Cetin H, Tarlak B, Bilgen AS (2012). Temporal bone fractures: evaluation of 77 patients and a management algorithm. Ulus Travma Acil Cerrahi Derg.

[15] Yetiser S, Hidir Y, Gonul E (2008). Facial nerve problems and hearing loss in patients with temporal bone fractures: demographic data. J Trauma.

[16] Gladwell M, Viozzi C (2008). Temporal bone fractures: a review for the oral and maxillofacial surgeon. J Oral Maxillofac Surg.

[17] Osetinsky LM, Hamilton GS 3rd, Carlson ML (2017). Sport injuries of the ear and temporal bone. Clin Sports Med.

[18] Zimmerman WD, Ganzel TM, Windmill IM, Nazar GB, Phillips M (1993). Peripheral hearing loss following head trauma in children. Laryngoscope.

